# Is the Depth of Invasion a Marker for Elective Neck Dissection in Early Oral Squamous Cell Carcinoma?

**DOI:** 10.3389/fonc.2021.628320

**Published:** 2021-03-12

**Authors:** Yassine Aaboubout, Quincy M. van der Toom, Maria A. J. de Ridder, Maria J. De Herdt, Berdine van der Steen, Cornelia G. F. van Lanschot, Elisa M. Barroso, Maria R. Nunes Soares, Ivo ten Hove, Hetty Mast, Roeland W. H. Smits, Aniel Sewnaik, Dominiek A. Monserez, Stijn Keereweer, Peter J. Caspers, Robert J. Baatenburg de Jong, Tom C. Bakker Schut, Gerwin J. Puppels, José A. Hardillo, Senada Koljenović

**Affiliations:** ^1^ Department of Pathology, Erasmus MC, University Medical Center Rotterdam, Rotterdam, Netherlands; ^2^ Department of Otorhinolaryngology and Head and Neck Surgery, Erasmus MC, University Medical Center Rotterdam, Rotterdam, Netherlands; ^3^ Department of Medical informatics, Erasmus MC, University Medical Center Rotterdam, Rotterdam, Netherlands; ^4^ Department of Oral and Maxillofacial Surgery, Erasmus MC, University Medical Center Rotterdam, Rotterdam, Netherlands; ^5^ Department of Dermatology, Erasmus MC, University Medical Center Rotterdam, Rotterdam, Netherlands

**Keywords:** oral cancer, squamous cell carcinoma of head and neck, depth of invasion, occult metastasis, elective neck dissection

## Abstract

**Objective:**

The depth of invasion (DOI) is considered an independent risk factor for occult lymph node metastasis in oral cavity squamous cell carcinoma (OCSCC). It is used to decide whether an elective neck dissection (END) is indicated in the case of a clinically negative neck for early stage carcinoma (pT1/pT2). However, there is no consensus on the cut-off value of the DOI for performing an END. The aim of this study was to determine a cut-off value for clinical decision making on END, by assessing the association of the DOI and the risk of occult lymph node metastasis in early OCSCC.

**Methods:**

A retrospective cohort study was conducted at the Erasmus MC, University Medical Centre Rotterdam, The Netherlands. Patients surgically treated for pT1/pT2 OCSCC between 2006 and 2012 were included. For all cases, the DOI was measured according to the 8^th^ edition of the American Joint Committee on Cancer guideline. Patient characteristics, tumor characteristics (pTN, differentiation grade, perineural invasion, and lymphovascular invasion), treatment modality (END or watchful waiting), and 5-year follow-up (local recurrence, regional recurrence, and distant metastasis) were obtained from patient files.

**Results:**

A total of 222 patients were included, 117 pT1 and 105 pT2. Occult lymph node metastasis was found in 39 of the 166 patients who received END. Univariate logistic regression analysis showed DOI to be a significant predictor for occult lymph node metastasis (odds ratio (OR) = 1.3 per mm DOI; 95% CI: 1.1–1.5, *p* = 0.001). At a DOI of 4.3 mm the risk of occult lymph node metastasis was >20% (all subsites combined).

**Conclusion:**

The DOI is a significant predictor for occult lymph node metastasis in early stage oral carcinoma. A NPV of 81% was found at a DOI cut-off value of 4 mm. Therefore, an END should be performed if the DOI is >4 mm.

## Introduction

Oral cavity cancer has a worldwide incidence of 350,000, with a male:female ratio of 2.1:1 (1). The 5-year survival rate is approximately 50% in Europe (2). Histologically, more than 90% of all oral cavity cancers are squamous cell carcinoma (OCSCC) (3). The most common risk factors for developing OCSCC are tobacco and alcohol consumption (4). In Southern Asia (India, Sri Lanka, China, and Thailand), the incidence of OCSCC is even higher due to the chewing of tobacco with or without betel quid (2). The estimated annual mortality in patients with OCSCC is 145,000 worldwide (5).

Factors that are known to contribute to a patients prognosis are tumor size, regional lymph node involvement and distance metastasis (TNM classification), tumor differentiation grade, perineural invasion (PNI), and lymphovascular invasion (LVI) (6). The treatment of choice is surgery with tumor resection and neck dissection in case of clinical lymph node involvement. An elective neck dissection in OCSCC patients is recommended if the risk of occult lymph node metastasis is >20% (7).

An END increases the disease-specific survival (DSS) and overall survival (OS) compared to watchful waiting (WW), supported by a therapeutic lymph node dissection when needed (8, 9). A neck dissection can be associated with several adverse effects such as edema, pain, and disability of the shoulder. The severity of these effects is often related to the extent of dissection; neck and shoulder discomfort is still reported even if the vital structures are well preserved (10, 11). Therefore, the current international consensus is that an END should only be performed if the risk of occult lymph node metastasis is >20%.

The DOI and sentinel lymph node biopsy are currently the best predictors for occult lymph node metastasis (12). Sentinel node biopsy has high accuracy for identifying occult lymph node metastasis (13–15). However, this accuracy is very dependent on experience and technical expertise, which makes the sentinel node biopsy procedure difficult for wide implementation (12).

The DOI is used as a marker for elective neck dissection (END) in a number of centers, including ours. However, there is no unanimous cut-off value, varying from 2 mm - 10 mm between the centers (16, 17). The lack of common definition and guidelines on how to measure DOI has led to this large variation. This shortcoming has been recently addressed by the 8^th^ edition of the cancer staging manual from the American Joint Committee on Cancer (AJCC) (18).

The aim of this study was to estimate a cut-off value of DOI for clinical decision making on END, by assessing the association of DOI and the risk of occult lymph node metastasis in early OCSCC.

## Method

### Study Design and Patients

A single-center retrospective cohort study was conducted at the Erasmus University Medical Center (Erasmus MC), Rotterdam, the Netherlands after Institutional Review Board approval (MEC-2016-751). Surgically treated patients with primary OCSCC (pT1 or pT2, based on the 8^th^ edition of the AJCC) and clinically negative lymph nodes (cN0) were identified from January 2006 until December 2012 (18). Clinical lymph node status was determined by palpation of the neck, and/or by imaging (ultrasound with fine-needle aspiration biopsy, CT, and/or MRI).

Exclusion criteria were a history of head and neck cancer, presence of synchronous oral cavity tumor, unreliable assessment of the DOI, and loss to follow-up.

All patient and tumor characteristics, except the DOI, were recorded from the patient files, including age, gender, tumor localization, cTNM, pTN, differentiation grade, perineural invasion (PNI), and lymphovascular invasion (LVI). Lymphovascular invasion was regarded as positive when appreciated in the tumor and/or in the cases of a positive lymph node (pN+).

Neck lymph node treatment (*i.e*., END or WW), follow-up (*e.g*., local recurrence, regional recurrence, and cause of death) were also recorded. Patients were divided into two groups based on the neck treatment: the END group and the WW group. All patients were followed for at least 5 years. Patients from the END group received clinical examination and ultrasonography when indicated. Patients in the WW group always underwent ultrasonography in the first 2 years of follow-up in addition to clinical examination. The frequency of the follow-up in the first 2 years was every 2–3 months, in the 3^rd^ year 4–6 months, and in the 4^th^ and 5^th^ years 6–12 months. If regional recurrence occurred, the side (ipsilateral or contralateral) was recorded.

### Measurement of the Depth of Invasion

The DOI was measured for all surgical specimens based on the hematoxylin and eosin slide. The DOI was defined and measured as a plumb-line from the basal membrane of the closest normal adjacent mucosa to the deepest point of invasion, in line with the recommendation from the 8^th^ edition of the AJCC (18).

All hematoxylin and eosin slides were collected from the Department of Pathology of the Erasmus University Medical Center and scanned by the NanoZoomer 2.0-HT slide scanner (Hamamatsu Photonics, Hamamatsu, Japan). Slides were reviewed by a head and neck pathologist (SK) using the NanoZoomer digital pathology (NDP) viewer 2.5.19 (Hamamatsu Photonics, Hamamatsu, Japan).

The patients were divided based on DOI into a group with DOI ≤4 mm and a group with DOI >4 mm, based on the DOI cut-off value >4 mm used at our institute.

### Statistical Analysis

Statistical analysis was performed using the IBM SPSS Statistics version 25 software. Patients’ characteristics between the two groups (DOI ≤ 4 mm DOI > 4 mm) were compared using student T-test for continuous variables and Chi-square test for categorical variables. Univariate logistic regression was performed to assess the correlation between predictor variables and occult lymph node status. A Receiver Operator Curve (ROC) was utilized to determine the optimal cut-off value for predicting occult lymph node metastasis using DOI, for all sub-sites combined. Follow-up was calculated from the date of surgery. Regional recurrence-free survival (*i.e.*, time until an isolated regional recurrence occurs; RRFS) and disease-specific survival (*i.e.*, time until death due to disease; DSS) were assessed by Kaplan–Meier analysis and log-rank test for the DOI ≤4 mm and >4 mm and for the WW and END in the DOI group ≤4 mm. The overall survival (*i.e.*, time until the death of patients; OS) was assessed by Kaplan–Meier analysis and log-rank test for the DOI ≤4 mm and >4 mm. Two-tailed statistical tests were performed. A p-value of less than 0.05 was considered statistically significant.

## Results

### Study Population

A total of 318 patients were seen in our hospital with pT1/pT2 OCSCC during the study period. Patients were excluded due to the following reasons: a history of head and neck tumor (n = 91), unreliable assessment of the depth of invasion (n = 3), loss to follow-up (n = 2). After exclusion, 222 patients were included for the final analysis, **Table 1**. Of the 222 patients included, the cN0 status was determined by both, clinical examination and imaging in 124 patients (55.9%), by clinical examination only in 51 patients (23%), and by imaging only in 42 patients (18.9%). For the remaining five patients (2.2%) no data was available.

**Table 1:** Patient and tumor characteristics.

	Number (n = 222)	%
**Gender**		
Male	138	62.2
Female	84	37.8
**Age (years)**		
Median (range)	64.5 (16.1–93.1)	
**pT status (8^th^ edition)**		
1	117	52.7
2	105	47.3
**Tumor diameter (cm)**		
Median (range)	1.5 (0.2–4)	
**Depth of invasion (mm)**		
Median (range)	4.48 (0.05–9.97)	
**Subsite**		
Tongue	128	57.6
Floor of mouth	65	29.3
Buccal mucosa	12	5.4
Retromolar trigone	7	3.2
Gingiva mandible*	7	3.2
Gingiva maxilla*	2	0.9
Lip	1	0.4
Hard palate	0	0.0
**Differentiation grade**		
Well	59	26.6
Moderate	149	67.1
Poor	14	6.3
**Perineural invasion**		
Yes	36	19.7
No	147	80.3
Unknown	39	
**Lymphovascular invasion**		
Yes	56	31.1
No	124	68.9
Unknown	42	
**Neck treatment**		
Ipsilateral END	146	65.8
Bilateral END	20	9.0
WW	56	25.2

*In this small group all patients had SCC arising from the gingiva. However, in five cases the tumor was extending to the adjacent floor of mouth, reaching the maximum DOI at that location.

### Depth of Invasion

Median DOI for all cases was 4.48 mm; mean was 4.8 mm with a standard deviation of 2.5 mm. In 97 cases the DOI was ≤4 mm and in 125 cases the DOI was >4 mm. Of all adverse histopathologic characteristics, only PNI was associated with DOI >4 mm (*p* = 0.001). The other adverse tumor characteristics such as differentiation grade and LVI were similar in both groups, **Table 2**.

**Table 2:** Comparison of patient and tumor characteristics for the two depth of invasion groups.

	DOI ≤ 4 mm	%	DOI > 4 mm	%	*p*-value*
**pT status (8^th^ edition)**					<0.001
1	89	91.8	28	22.4	
2	8	8.2	97	77.6	
Tumor diameter**	1.23 ± 0.69		1.94 ± 0.83		<0.001
DOI**	2.47 ± 0.95		6.62 ± 1.75		<0.001
**Subsite**					0.670
Tongue	59	60.8	69	55.2	
Floor of mouth	28	28.9	37	29.6	
Buccal mucosa	3	3.1	9	7.2	
Retromolar trigone	3	3.1	4	3.2	
Gingiva mandible	3	3.1	4	3.2	
Gingiva maxilla	0	0.0	2	1.6	
Lip	1	1.0	0	0.0	
Hard palate	0	0.0	0	0.0	
**Differentiation grade**					0.259
Well	31	32.0	28	22.4	
Moderate	61	62.8	88	70.4	
Poor	5	5.2	9	7.2	
**Perineural invasion**					0.001
Yes	6	8.2	30	27.3	
No	67	91.8	80	72.7	
Unknown	24		15		
**Lymphovascular invasion**					0.10
Yes	7	10.4	16	15.1	
No	60	89.6	90	84.9	
Unknown	30		19		

*Chi-square test for categorical data, unpaired T-test for numeric data.

**Expressed as mean ± SD.

### Elective Neck Dissection *Versus* Watchful Waiting

Thirty-nine patients of the 166 patients treated with an END had occult lymph node metastasis. The DOI of all patients was categorized into whole mm (0 mm < DOI ≤ 1 mm, 1 mm < DOI ≤ 2 mm, *etc*), **Table 3**. A separate analysis was performed for 128 patients with SCC of the tongue, **Table 4**.

**Table 3:** Association between depth of invasion and occult lymph node metastasis.

DOI (mm)	Total patients (n)	pN0 (n)	pN+** n (%)	Cut-off value (mm)	Sens* (%)	Spec* (%)	PPV* (%)	NPV* (%)
1 (0 < DOI ≤ 1)	2	2	0 (0)	>1	100	2	24	100
2 (1 < DOI ≤ 2)	6	6	0 (0)	>2	100	6	25	100
3 (2 < DOI ≤ 3)	24	20	4 (17)	>3	90	22	26	88
4 (3 < DOI ≤ 4)	21	15	6 (29)	>4	74	34	26	81
5 (4 < DOI ≤ 5)	26	20	6 (23)	>5	59	50	26	80
6 (5 < DOI ≤ 6)	16	14	2 (12)	>6	54	61	30	81
7 (6 < DOI ≤ 7)	24	21	3 (12)	>7	46	77	38	82
8 (7 < DOI ≤ 8)	16	9	7 (44)	>8	28	84	35	79
9 (8 < DOI ≤ 9)	15	9	6 (40)	>9	13	91	31	77
10 (9 < DOI ≤ 10)	16	11	5 (31)	>10	0	100	#N/B	77

*Sensitivity, specificity, PPV, and NPV were calculated using the upper limit of the category as a cut-off.

**Percentage is based on the pN+ per categorized DOI (mm).

**Table 4:** Association between depth of invasion and occult lymph node metastasis in tongue.

DOI (mm)	Total patients (n)	pN0 (n)	pN+** n (%)	Cut-off value (mm)	Sens* (%)	Spec* (%)	PPV* (%)	NPV* (%)
1 (0 < DOI ≤ 1)	4	4	0 (0)	>1	100	4	31	100
2 (1 < DOI ≤ 2)	12	10	2 (17)	>2	95	16	33	88
3 (2 < DOI ≤ 3)	23	16	7 (30)	>3	77	34	34	77
4 (3 < DOI ≤ 4)	20	12	8 (40)	>4	56	47	32	71
5 (4 < DOI ≤ 5)	19	14	5 (26)	>5	44	63	34	72
6 (5 < DOI ≤ 6)	8	7	1 (12)	>6	41	71	38	73
7 (6 < DOI ≤ 7)	15	12	3 (20)	>7	33	84	48	74
8 (7 < DOI ≤ 8)	7	4	3 (43)	>8	26	89	50	73
9 (8 < DOI ≤ 9)	9	4	5 (56)	>9	13	93	45	71
10 (9 < DOI ≤ 10)	11	6	5 (45)	>10	0	100	#N/A	70

*Sensitivity, specificity, PPV, and NPV were calculated using the upper limit of the category as a cut-off.

**Percentage is based on the pN+ per categorized DOI (mm).

**Figure 1:**
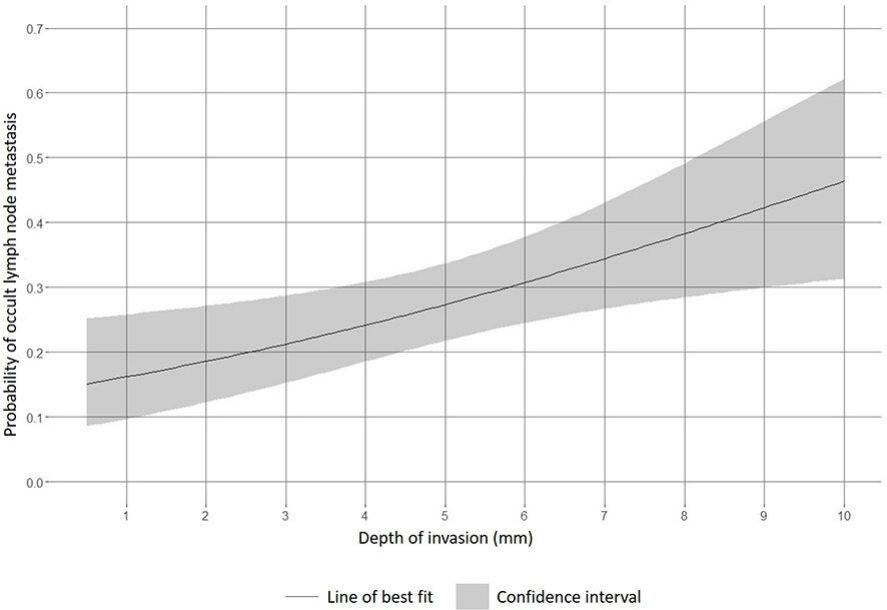
Association between depth of invasion and occult lymph node metastasis.

**Figure 1** shows predictions from a logistic regression analysis. This leads to a cut-off value of 4.3 mm, considering the 20% risk (NPV = 80%) (7). In the logistic regression analysis for the tongue population, the risk of 20% (NPV = 80%) is reached between 3 mm and 4 mm.

### Predictors for Occult Lymph Node Metastasis

Univariate logistic regression analysis showed depth of invasion (OR = 1.3 per mm DOI; 95% CI: 1.1–1.5, *p* = 0.001) and tumor diameter (OR = 2.0; 95% CI: 1.3–3.1, *p* = 0.002) as predictors for occult lymph node metastasis. Perineural invasion (*p* = 0.204) and differentiation grade (*p* = 0.194) were non-predictors for occult lymph node metastasis.

### Follow-Up

The mean follow-up was 67 ± 34 months, ranging from 0.2 to 156 months. No difference was found in the duration of follow-up between the DOI ≤4 mm and >4 mm, *p* = 0.969 (66.7 ± 33.5 months; 66.5 ± 34.9 months, respectively).

No difference was found between the groups DOI ≤ 4 mm and > 4 mm in local recurrence, and distant metastasis. Local recurrence occurred in 19 patients, 8 patients (8.2%) in the group DOI ≤ 4 mm and 11 patients (8.8%) in the group DOI > 4 mm, *p* = 1.0. Distant metastasis occurred in 12 patients, 6 patients (6.2%) in the group DOI ≤ 4 mm and 6 patients (4.8%) in the group DOI > 4 mm, *p* = 0.878.

Regional recurrence was also analyzed per DOI group (≤4 mm *versus >*4* mm*) and per type of treatment (WW *versus* END), **Table 5**. Regional recurrence occurred in 15 patients (15.5%) in the group DOI ≤4 mm and in 12 patients (9.6%) in the group DOI >4 mm, *p* = 0.263.

**Table 5:** Regional recurrence for the two depth of invasion groups.

DOI ≤ 4 mm					DOI > 4 mm			
	Number of patients (n = 97)	Regional Recurrence (n)			Number of patients (n = 125)	Regional Recurrence (n)		
		2 yr	5 yr	Total		2 yr	5 yr	Total
**WW**	44 (45.4%)	8	3	11 (25%)	12 (9.6%)	1	1	2 (16.7%)
**END**	53 (54.6%)			4 (7.7%)	113 (90.4%)			10 (8.8%)
**pN0**	43 (81.1%)	2	1		84 (74.3%)	3	3	
**pN+**	10 (18.9%)	0	1		29 (25.7%)	3	1	

In the WW group, regional recurrence was seen in 13 patients (23.2%) (11 in the group DOI ≤4 mm and two in the group DOI >4 mm) and 14 patients (8.4%) in the END group (four in the group DOI ≤4 mm and 10 in the group DOI >4 mm), *p* = 0.007.

In this END group, in nine of 14 cases regional recurrence was contralateral (tumor subsite: tongue six, floor of mouth two, and retromolar trigone one). In the remaining five cases the regional recurrence was ipsilateral, four in a level which was not included in the END, one in the level that was included.

Regional recurrence-free survival was similar for a DOI ≤4 mm and a DOI >4 mm (5-year RRFS 86.0 *vs* 90.1%, logrank test p = 0.317).

Disease specific survival was similar for a DOI ≤4 mm and a DOI >4 mm (both 5-year DSS 89.1 *vs* 91.3%, log-rank test p = 0.605).

Overall survival was similar for a DOI ≤4 mm and a DOI >4 mm (5-year OS 73.6 *vs* 70.1%, log-rank test p = 0.527).

The differences in RRFS and DSS were calculated between WW and END only for the group DOI ≤4 mm, because in the group DOI >4 mm the number of patients with WW was not sufficient for statistical analysis.

**Figure 2:**
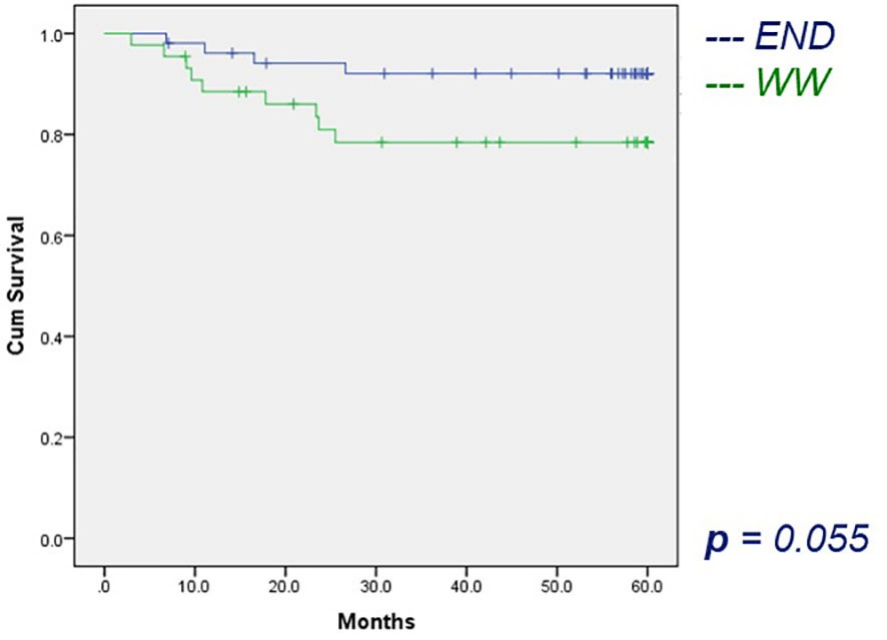
The 5-year regional recurrence-free survival.

For the group DOI ≤4 mm, the RRFS for patients with an END compared to those with WW was not different (5-year RRFS 92.2 *vs* 78.4%, log-rank test p = 0.055), **Figure 2**.

For the DOI ≤4 mm, the DSS was similar for the END and WW (5-year DSS 94.3 *vs* 82.6%, log-rank test p = 0.097).

## Discussion

Several studies report the DOI as a predictor of occult lymph node metastasis, and it is used as a criterion to decide on END in early OCSCC (19–26).

However, large differences exist between studies in regard to the definition and reliable measurement of the DOI and in the number of cases included from different subsites. This makes comparison of the results between studies unreliable.

The lack of consensus on the DOI cut-off value for the clinical decision on END is caused by the fact that it is used interchangeably with tumor thickness (TT) in different studies (16, 17, 19, 20, 27, 28). The DOI is considered a better prognostic factor than TT because it compensates for exophytic or ulcerative tumors (28). The 8^th^ edition of the AJCC guideline, published in January 2017, provides a clear definition of the DOI (*i.e*., the distance between the basal membrane of normal adjacent mucosa and the deepest point of tumor invasion) (18). Therefore, many studies are outdated (9, 19, 28–30). Moreover, the studies published after the release of the 8th edition of the AJCC show large variances. A number of studies do not confirm the DOI cut-off value of 4 mm. For instance, Faisal et al. showed 10 mm DOI cut-off value for decision making on END, Tam et al. showed 7.25 mm, and Kozak et al. did not specify another DOI cut-off value (23, 24, 31). On the other hand, van Lanschot et al. confirmed the DOI cut-off value of 4 mm, and Brockhoff et al. calculated DOI cut-off values for most subsites (*i.e.*, tongue = 2mm, floor of mouth = 3mm, and Proc alv/hard palate = 4mm) (20, 22).

The strength of the current study is that the DOI was measured for all cases, according to the current AJCC guideline, on digital H&E slides with high precision. In order to have comparable data, it would be desirable that in future studies the DOI is used and that the conclusions of already published studies based on TT are reassessed based on the DOI.

It is known that the frequency of occult lymph node metastasis differs per OCSCC subsite. It has been reported that occult lymph node metastasis is present in 20–30% of the cases for tongue cancer, 41.7% for the floor of mouth, and 15.4% for the buccal mucosa (20, 32). Therefore, the DOI cut-off value should be determined per subsite. The limited number of cases per subsite included in this study did not allow this analysis.

Aside from the DOI, other tumor characteristics like diameter, differentiation grade, worst pattern of invasion, perineural invasion, and tumor budding can also be associated with occult lymph node metastasis (33–36). In this study, it was not possible to confirm the other tumor characteristics because the multivariate analysis was not performed due to the incomplete pathology reporting between 2006 and 2012. Data on LVI, PNI, and tumor diameter were sometimes missing. Besides, margin status was often not annotated exactly. Instead of numerical values, there was only a description of margins (*e.g*., radical, free of tumor). The previously published study on this subject by our group involved a relatively recent cohort (2013–2018), in which our protocol for END was based on the DOI (>4 mm = END). On contrary, in the current study an older cohort was involved for which the guideline for END was based on either DOI >5 mm or tumor diameter >1.0 cm. Moreover, for the old cohort the reliable data for LVI, PNI, tumor diameter and margin status were missing and therefore not further analyzed and compared with the newer cohort. Finally, the patient outcome (locoregional recurrence and survival) in the previously published study may be influenced by the fact that our institute started with intra-operative assessment of resection margins in 2013 (22, 37, 38).

However, it was shown that a predictive model for occult lymph node metastasis including all the tumor characteristics is the best approach (39). Objective methods for predicting occult lymph node metastasis are being investigated, like gene-expression profiling or molecular markers (40–43).

In this study, we showed that the DOI is a significant predictor for occult lymph node metastasis (*p* = 0.001) in OCSCC. Therefore, the DOI can be regarded as a parameter for decision making on END. At our institute, the DOI cut-off value >4 mm is used, based on the National Comprehensive Cancer Network (NCCN) guideline (12). Here we confirm with a NPV of 81% the DOI cut-off value >4 mm for decision making on END.

We showed that performing an END in patients with an DOI ≤4 mm had no significant effect on the 5-year DSS compared to WW (94.3 *vs* 82.6%, log-rank test *p* = 0.097). The strength of this study is that this analysis was possible because of the large number of patients treated with an END in the group with a DOI ≤4 mm. In this group, the RRFS reached near significance (*p* = 0.055) for END, when compared to WW. For the group DOI >4 mm, the difference in DSS and RRFS could not be calculated because the number of patients was not sufficient for statistical analysis.

Despite the fact that END was performed, regional recurrence occurred in 8.4% of patients (14 of 166). The recurrences were either ipsilateral and mostly at a neck level that was not included in the END (5), or contralateral (9) to END side. The effectiveness of END is shown by the fact that only one patient had a regional recurrence at a level that was included in the END.

Most authors base their decision on END according to 20% (NPV 80%) risk of occult lymph node metastasis (19, 20, 22–26). The origin of this risk cut-off value is the publication of Weiss et al. in 1994 (7). In this study, the decision for intervention was determined by the side effects of surgery (END) and radiotherapy at that time. It may be assumed that nowadays, 25 years later, the treatment modalities have substantially improved. Therefore, we suggest that a risk lower than 20% should be taken into consideration when deciding on END. This of course, should only be done in agreement with patients, based on the clear information on both, side effects of the END and the risk of occult lymph node metastasis.

## Data Availability Statement

The raw data supporting the conclusions of this article will be made available by the authors, without undue reservation.

## Ethics Statement

The studies involving human participants were reviewed and approved by the Medisch Ethische Toetsings Commissie Erasmus MC (MEC-2016-751). The patients/participants provided their written informed consent to participate in this study.

## Author Contributions

YA designed the study, performed the depth of invasion measurements with SKo, carried out the retrospective database study, and drafted the manuscript. QT designed the study, performed the depth of invasion measurements with SKo, and carried out the retrospective database study. MR was responsible for the statistical analysis of data. MH and BS were responsible for the collection and scanning of histopathologic material and revised the manuscript critically for important intellectual content. CL, EB, MN, IH, HM, RS, AS, DM, SKe, PC, and RB revised the manuscript critically for important intellectual content. TS anticipated the design of the study and revised the manuscript critically for important intellectual content. GP designed the study, supervised the research group, and revised the manuscript critically for important intellectual content and gave the final approval of the version to be published. JH designed, drafted, and supervised this study and gave the final approval of the version to be published. SKo designed, drafted, and supervised this study, was mainly responsible for the depth of the invasion measurements, and gave the final approval of the version to be published. All authors contributed to the article and approved the submitted version.

## Funding

We thank the Dutch Cancer Society (106467-Optimizing surgical results for oral squamous cell carcinoma by intra-operative assessment of resection margins using Raman spectroscopy) and Eurostars (12076-RA-SURE) for the financial support.

## Conflict of Interest

The authors declare that the research was conducted in the absence of any commercial or financial relationships that could be construed as a potential conflict of interest.

## Abbreviations

DOI, depth of invasion; OCSCC, oral cavity squamous cell carcinoma; END, elective neck dissection; WW, watchful waiting; OR, odds ratio; MC, Medical Center; AJCC, American Joint Committee on Cancer; NCCN, National Comprehensive Cancer Network; H&E, hematoxylin and eosin; PNI, perineural invasion; LVI, lymphovascular invasion; OS, overall survival; DSS, disease-specific survival; RRFS, regional recurrence-free survival; TT, tumor thickness; TNM, Tumor, lymph nodes, metastasis (according to the TNM Classification of Malignant Tumors).
